# Integrated analysis of transcriptome sequencing and metabolomics provides insights into the molecular response of *Solanum tuberosum* cv. Cooperation 88 to Potato virus S

**DOI:** 10.3389/fmicb.2026.1828392

**Published:** 2026-07-15

**Authors:** Kuo Wu, Xia Liu, Yu Li, Yongdui Chen, Qinglan Zhao, Zhongkai Zhang, Yanli Yang

**Affiliations:** 1 College of Plant Protection, Yunnan Agricultural University, Kunming, Yunnan, China; 2 Biotechnology and Germplasm Resources Institute, Yunnan Academy of Agricultural Sciences; Yunnan Provincial Key Lab of Agricultural Biotechnology, Kunming, Yunnan, China

**Keywords:** metabolomics, molecular response, Potato virus S, *Solanum tuberosum* cv. Cooperation 88, transcriptome

## Abstract

**Introduction:**

Potato virus S (PVS), is an important member of the genus *Carlavirus* in the family *Betaflexiviridae*, is a significant pathogen in potatoes worldwide. Previous studies have found it to be the most frequently detected virus in potatoes in China. However, research data on the response mechanisms of potatoes to PVS remain extremely scarce.

**Methods:**

Here, comparative transcriptomics and metabolomics were performed on potato (*Solanum tuberosum* cv. Cooperation 88) leaves to elucidate the response mechanisms underlying the infection of Potato virus S to *Solanum tuberosum* cv. Cooperation 88 at the molecular level.

**Results:**

A total of 588 significantly differentially expressed genes (SDEGs) were identified post-infection, including 161 upregulated genes and 427 downregulated genes, primarily enriched in biological processes such as the MAPK signaling pathway, plant hormone signal transduction, phenylpropanoid metabolism, and DNA replication. Metabolomic analysis revealed 1,313 and 880 differential metabolites detected in positive and negative ion modes, respectively, with flavonoids showing a significant accumulation trend. Integrated analysis indicated that the phenylpropanoid metabolic pathway (particularly the flavonoid biosynthesis pathway) plays a central regulatory role in antiviral responses. Real-time quantitative PCR validation further confirmed that the expression patterns of key genes (e.g., PAL, C4H, 4CL) were positively correlated with metabolite accumulation.

**Conclusion:**

The results suggest that the flavonoid biosynthesis pathway in potatoes plays a key role in responding to PVS infection. This study not only helps to deeply understand the interaction mechanism between PVS and potatoes, but also promotes the selection and utilization of disease-resistant varieties.

## 1 Introduction

Potato (*Solanum tuberosum*), recognized as the world’s fourth most significant staple crop after rice, wheat, and maize, plays a pivotal role in global food security and agricultural sustainability. China leads global production, contributing about one-quarter of the total. However, the potato industry has long faced dual threats from biotic and abiotic stresses, among which Potato virus S (PVS) is one of the most prevalent viral pathogens in major production regions like Yunnan Province, which accounts for 10% of China’s total potato hectare and where PVS infection rates can reach 30–50% (Zhang et al., 2015; Du et al., 2020). PVS spreads through aphids (*Myzuspersicae*) in a non-persistent manner and causes symptoms such as leaf mottling, curling, and necrosis, resulting in a significant decline in tuber yield and severely restricting the industry’s profitability (Zhang and Li, 2001; Zhang et al., 2022). Current PVS control relies on virus-free seed potatoes and chemical control of aphids, but biological control is slow to take effect and chemical control is restricted by environmental protection policies. Breeding of disease-resistant varieties is the fundamental solution; however, the scarcity of PVS-resistant germplasm, long-distance seed potato transport exacerbating virus spread, and limited research on PVS resistance mechanisms at the molecular level highlight the urgent need to elucidate host-pathogen interactions to provide a theoretical foundation for disease-resistant breeding.

The interaction between plants and viruses is a dynamic arm race of “virus hijacking host – plant activating defense”: viruses destroy host cells through replication and spread, while plants employ multi-layered defenses such as RNA silencing, effector-triggered immunity (ETI), hormone signaling pathways, and secondary metabolic reprogramming to limit viral infection (Ding and Voinnet, 2007; Mandadi and Scholthof, 2013; Xu et al., 2024; Boller and He, 2009; Jiang et al., 2025). Among these, flavonoid metabolism, as a key downstream branch of the phenylpropanoid pathway, has strong antioxidant activity due to its multiple hydroxyl groups (-OH), enabling efficient scavenging of virus-induced reactive oxygen species (ROS) and mitigating oxidative damage, making it one of the core secondary metabolic pathways for plant stress resistance (Yadav V. et al., 2020; Liu et al., 2021). Studies have confirmed that flavonoids are involved in processes mulberry resistance to bacterial wilt (Dai et al., 2019), tomato resistance to Tomato Yellow Leaf Curl Virus (TYLCV) (Sade et al., 2015), and watermelon resistance to cucumber green mottle mosaic virus (Liu et al., 2023), and enhanced phenylpropanoid metabolism in tobacco has been shown to improve resistance to tobacco mosaic virus (TMV) (Shadle et al., 2003). These studies suggest that flavonoid metabolism may be a common mechanism for plant resistance to viruses. However, the regulatory mechanism of flavonoids in the interaction between PVS and potatoes remains unclear.

Although transcriptomic and metabolomic analyses have been employed to elucidate plant disease resistance mechanisms (Sade et al., 2015; Mansfeld et al., 2017; Maridueña-Zavala et al., 2025a,b). They are also an important means to reveal the molecular responses of potato-pathogen interactions, for example, Baebler et al. (2020) and Li et al. (2025) compared molecular responses of Susceptible and resistant potato varieties to potato virus Y and Early Blight; Kogovšek et al. (2016) performed an integrative study that links changes in the metabolome and gene expression in potato leaves inoculated with the mild PVY^N^ and aggressive PVY^NTN^ isolates, the results indicated that primary metabolism, phenylpropanoids and antioxidant pathways in potato leaves are regulated. There remains a significant gap in integrated transcriptomic-metabolomic analysis of PVS infection. Current studies have yet to systematically reveal the cascade regulatory network of “PVS infection-host gene expression reprogramming-characteristic metabolite accumulation.” While PVS infection alone has relatively limited impact on potato yield, clarifying the molecular basis of PVS-potato interaction holds significant importance for advancing plant-virus interaction theory. This study established an infection system by artificially inoculating PVS into the main cultivar *Solanum tuberosum* cv. Cooperation 88(Co-88) in Yunnan, combining transcriptome sequencing (RNA-seq), extensive untargeted metabolomic analysis, and RT-qPCR validation to systematically analyze the gene expression and metabolite changes in potatoes responding to PVS. The research focuses on addressing: (1) the core transcriptional and metabolic changes in potatoes under PVS infection; (2) whether flavonoid metabolism plays a dominant role in the resistance process against PVS; and (3) how key genes and metabolites synergistically regulate resistance. The study aims to elucidate the molecular mechanisms of potato resistance to PVS, providing molecular markers and theoretical foundations for breeding disease-resistant varieties, as well as offering new insights into the regulation of secondary metabolism in plant-virus interactions.

## 2 Materials and methods

### 2.1 Plant material and growth conditions

*Solanum tuberosum* cv. Cooperation88 (Co-88), a main cultivated variety in Yunnan province, was used as the experimental material in this study. Seedlings were kindly provided by our laboratory at Institute of Biotechnology and Germplasm Resources, Yunnan Academy of Agricultural Sciences, located in Kunming City, Yunnan Province, China. Each seedling was transplanted into a plastic pot with an upper diameter of 40 cm, a lower diameter of 20 cm, and a depth of 20 cm, filled with approximately 8 L of nutrient soil. Plants were cultivated in an environmentally controlled greenhouse at the laboratory under a temperature of 25 ± 2 °C, a relative humidity of 75%, and a photoperiod of 14 h light/10 h dark with a light intensity of 2000Lx.

### 2.2 Pathogen inoculation and PVS testing

The isolate of Potato virus S (PVS) infecting *S. tuberosum* was obtained from our laboratory collection (Zhang et al., 2022). The virus inoculation procedure was performed following the method reported by Hu et al. (2023), with minor modifications. Three-week-old, virus-free *S. tuberosum* seedlings, grown in the greenhouse facilities at the Yunnan Academy of Agricultural Sciences (YAAS), were selected as host material.

For inoculation, the surface of a healthy leaf was first cleaned using wet sterile cotton. Autoclaved carborundum powder was then evenly dusted onto the leaf surface to facilitate viral entry. Two leaves per plant were designated as inoculation sites. A virus-infected leaf was ground in an inoculation buffer, and the resulting extract was gently rubbed onto the prepared leaves using a sterilized cotton swab. Ten minutes after inoculation, the carborundum abrasive was carefully rinsed off the leaves with sterile water. The inoculated seedlings were subsequently transferred to a screened greenhouse room for cultivation. Leaves inoculated with sterile water alone served as negative controls (CK). A total of 20 leaves were inoculated with the PVS isolate, including 10 control leaves. All plants were maintained in the YAAS greenhouse under standard conditions throughout the experiment.

Symptom observation and analysis were conducted on the 7th and 14th days post-inoculation (dpi). The number of successful infection sites was recorded to calculate the infection incidence (%) on the 7th dpi. Following this initial assessment, all inoculated plants were retained in the greenhouse for further analysis. Subsequent confirmation of virus infection was performed using both serological assays and electron microscopy.

### 2.3 Transcriptome sequencing and data analysis

Transcriptome analysis of Co-88 infected with Potato virus S (PVS) was performed at 14 days post-infection (dpi), with healthy plants serving as negative controls (designated as V and CK). For each treatment group, three biological replicates were collected. Leaf samples were harvested from the third leaf below the apex. Immediately after collection, all samples were flash-frozen in liquid nitrogen and stored at −80 °C until further processing.

Total RNA was extracted using TRIzol reagent (Invitrogen, Carlsbad, CA, United States) following the manufacturer’s protocol. RNA concentration, purity, and integrity were assessed using a NanoDrop spectrophotometer (Thermo Fisher Scientific, Waltham, MA, United States) and agarose gel electrophoresis. Samples meeting quality criteria were submitted to OE Biotech Co., Ltd. (Shanghai, China) for transcriptome sequencing on the Illumina HiSeq 2,500 platform.

Raw sequencing reads were initially evaluated for quality using FastQC. Subsequently, adapter-containing sequences, reads with over 10% ambiguous bases (*N*), and those with an average Phred quality score below 20 (Q20) were filtered out to generate high-quality clean reads. All downstream analyses were conducted using these cleaned datasets. The six RNA-seq libraries (three biological replicates each for the V and CK groups) were pooled for *de novo* transcriptome assembly using Trinity software (version 2.11.0). The resulting unigenes were functionally annotated against multiple public databases, including the NCBI non-redundant protein (NR) database, Gene Ontology (GO), and Kyoto Encyclopedia of Genes and Genomes (KEGG).

Gene expression levels were quantified as fragments per kilobase of transcript per million mapped reads (FPKM). Sample correlation analysis was performed using variance-stabilized transformation (VST) values, while principal component analysis (PCA) was conducted on regularized log-transformed (rlog) read counts. Differential gene expression analysis was carried out using the DESeq2 R package. Transcripts exhibiting an adjusted *p*-value (padj) < 0.05 and an absolute log_2_ fold change ≥1 were classified as differentially expressed genes (DEGs). To elucidate the potential biological functions of the identified DEGs, Gene Ontology (GO) enrichment analysis was performed using the top GO package, and pathway enrichment analysis was conducted using the cluster Profiler R package.

### 2.4 Widely untargeted metabolome analysis

At 28 dpi, leaves of Co-88 were sampled for secondary metabolite profiling. Plants infected with PVS and healthy controls were labeled as PVS and CK, respectively. Sampling procedures matched those used for transcriptome analysis, and six biological replicates were prepared per treatment. Metabolite extraction and LC–MS/MS analysis were performed by Novogene Science and Technology Co., Ltd. (Beijing, China).

Approximately 100 mg of leaf tissue was ground in liquid nitrogen and homogenized in pre-chilled 80% methanol with thorough vortexing. Samples were incubated on ice for 5 min and centrifuged at 15,000 × g, 4 °C for 20 min. An aliquot of the supernatant was diluted with LC–MS-grade water to a final methanol concentration of 53%, transferred to fresh tubes, and centrifuged again under identical conditions. The final supernatants were injected into an LC–MS/MS system for analysis (Dunn et al., 2011).

Raw data files acquired via ultra-high-performance liquid chromatography–tandem mass spectrometry (UHPLC–MS/MS) were processed using XCMS for feature detection, peak alignment, and quantification (Want et al., 2010). Metabolites were identified by matching adduct ion patterns against a proprietary high-resolution spectral database (Novo Met DB), allowing a mass tolerance of 10 ppm. Signals detected in blank samples were removed as background. Quantitative values were normalized using the formula:


$$\begin{array}{l} \text{Relative peak area}=(\mathrm{Raw}\;\text{sample value})/\\ \;\;\;\;\;\;[(\mathrm{Sum}\;\text{of sample values})/(\mathrm{Sum}\;\text{of}\;\mathrm{QC1}\;\text{values})] \end{array}$$

Metabolites showing a coefficient of variation (CV) > 30% across QC samples were excluded. All data processing was conducted on a CentOS 6.6 (Linux) operating system using R and Python; detailed software versions are listed in the accompanying README file.

Differentially accumulated metabolites (DAMs) were identified based on a variable importance in projection (VIP) score ≥ 1, FC > 1.5. Functional annotation was performed using the KEGG Compound database to associate DAMs with metabolic pathways potentially linked to *S. tuberosum* resistance against PVS infection.

### 2.5 Validation of gene expression from transcriptome data

To validate the reliability of the Illumina RNA-seq results, Reverse Transcription-quantitative PCR (RT-qPCR) was performed for 10 differentially expressed genes (DEGs) associated with flavonoid metabolism. The leaf samples used for RT-qPCR were identical to those used in the transcriptome analysis and included both PVS-infected (V) and control (CK) groups. Each treatment was represented by three biological replicates.

Primer sequences for the selected DEGs were designed using Primer Premier 5 software based on the assembled transcriptome data (Supplementary Table S1). Total RNA was extracted using the Steady Pure Plant RNA Extraction Kit (Accurate Biology, Changsha, China), and genomic DNA was removed during purification. Reverse transcription was carried out using the Evo M-MLV RT Kit with gDNA Clean for qPCR (Accurate Biology, Changsha, China) to synthesize complementary DNA (cDNA).

RT-qPCR was performed on an ABI real-time PCR detection system (Applied Biosystems, California, United States) using the Evo M-MLV One Step RT-qPCR Kit (Accurate Biology, Changsha, China), strictly following the manufacturers’ protocols. Each reaction had a total volume of 20 μL and was run under the following thermal cycling conditions: initial denaturation at 95 °C for 30 s, followed by 40 cycles of 95 °C for 5 s; 60 °C for 30 s. Each sample was analyzed with three technical replicates.

Gene expression levels were calculated using the 2^^−ΔΔCt^ method, with the *Actin* gene of *S. tuberosum* serving as the internal reference. Data are presented as mean ± SD and statistically significant differences were determined using Microsoft Excel.

## 3 Results

### 3.1 Differences in *Solanum tuberosum* varieties following PVS infection

**Figure 1:**
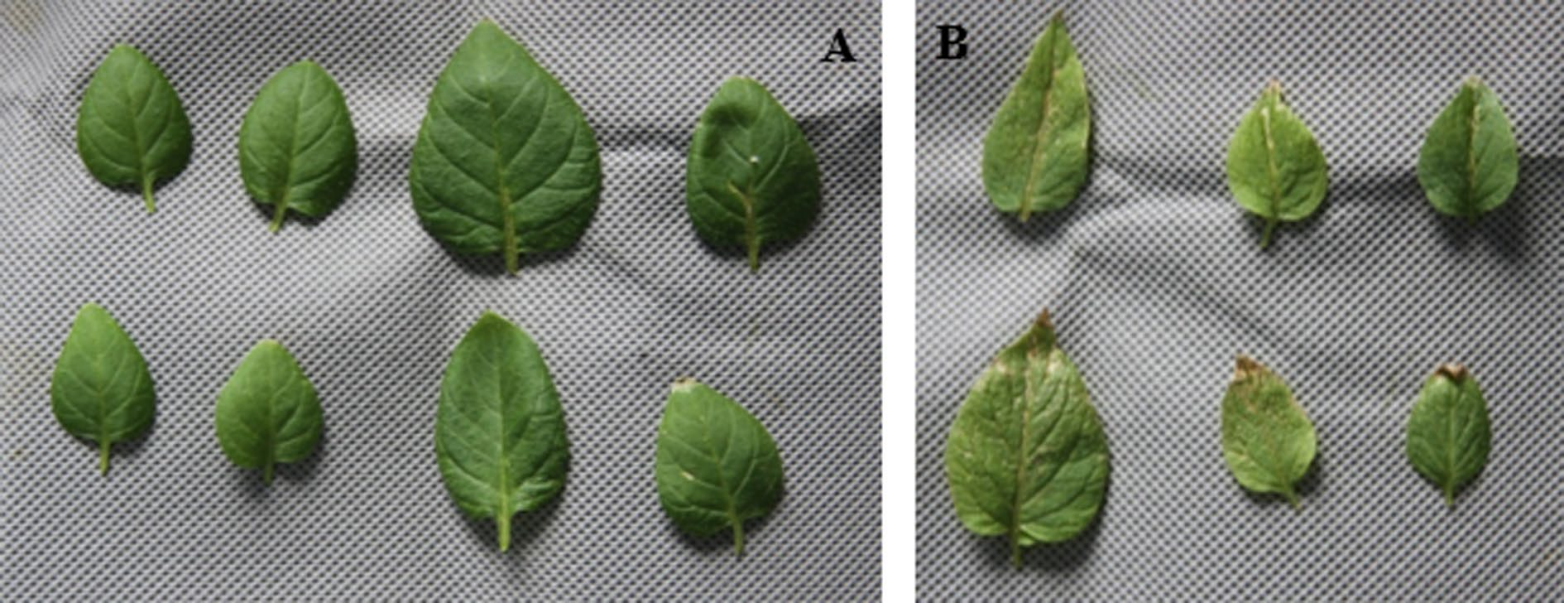
Potato material (Cooperation 88) in greenhouse. **(A)** CK; **(B)** PVS inoculation.

At 14 days post-infection (dpi), no significant differences in infection incidence were observed among the leaves of various *S. tuberosum*. All inoculated seedlings continued to be maintained in culture. Infected leaves exhibited light-colored mottling (Figure 1).

**Figure 2:**
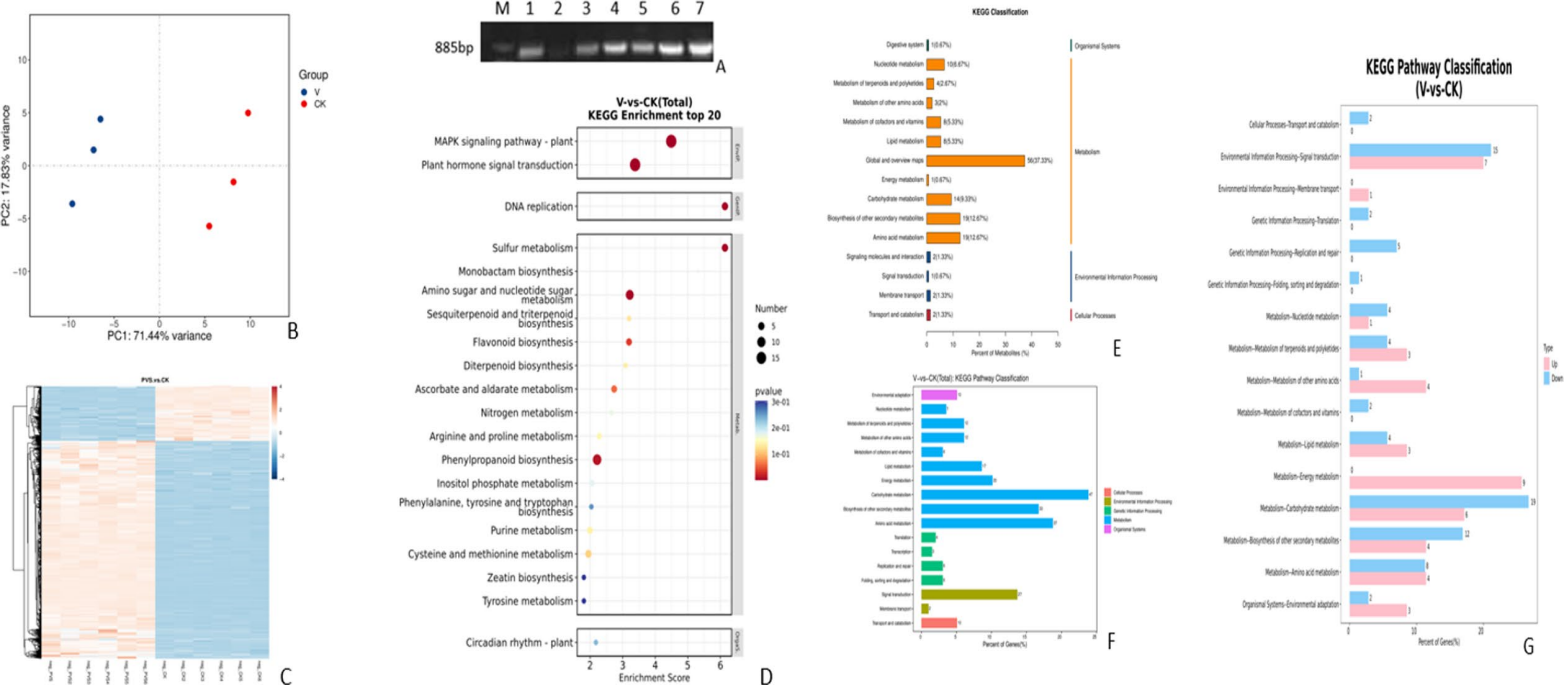
Transcriptome sequencing results of PVS-infected samples. **(A)** Electrophoretic gel image of PVS coat protein (cp) gene amplified by RT-PCR (M: Marker2000; 1, Positive; 2, Negative; 3–7 PVS coat protein gene); **(B)** PCA analysis of the expression of unigenes for V vs. CK; **(C)** Heatmap of DAGs in V and CK leaf. **(D)** Top 20 enriched KEGG pathways of DEGs and possible pathways related with the virus resistance of *S. tuberosum*; **(E)** DAMs categorized KEGG databases; **(F)** KEGG enrichment of DEGs function; **(G)** Category and number of DAMs; (“V” indicates samples inoculated with PVS).

To validate the success of viral inoculation, the transcriptional expression levels of the PVS coat protein (CP) gene were detected in infected leaves. Reverse transcription PCR (RT-PCR) analysis confirmed the presence of the *CP* gene in the sampled leaves following inoculation. The results demonstrated that every biological replicate designated for RNA-seq contained detectable PVS CP gene (Figure 2A).

The screening and utilization of disease-resistant genes represent a highly effective and environmentally sustainable strategy for controlling plant diseases and enhancing breeding efficiency. Previous field surveys of PVS distribution in various *S. tuberosum* cultivars revealed widespread occurrence throughout the main potato-growing regions of Yunnan Province, with only a limited number of immune varieties identified. Such field-based assessments of disease resistance can offer valuable guidance for the deployment of resistant cultivars. However, field conditions are often influenced by environmental variables and agronomic practices, which may obscure the intrinsic genetic resistance of plant varieties. When feasible, genetic resistance remains the preferred approach for durable disease management. Artificial inoculation systems, typically conducted under controlled environmental conditions with uniform plant physiology, allow for a more accurate evaluation of host resistance traits (Gevens et al., 2006). In the present study, an artificial inoculation method was employed to identify resistance-related pathways against PVS, providing a reliable foundation for developing effective control strategies for PVS in *S. tuberosum*.

### 3.2 Illumina sequencing and functional annotation of unigenes

To investigate the potential resistance mechanisms of Co-88 in response to Potato virus S (PVS) infection, three independent biological replicates from the PVS-responsive group at 14 days post-inoculation (dpi) were selected for transcriptome analysis. A total of 6 cDNA libraries (three biological replicates each for the V [PVS-infected] and CK [mock-inoculated] groups) were constructed and pooled for sequencing. Each sample yielded approximately 5.93–7.08 Gb of clean reads, with a Q30 score exceeding 91%, indicating high sequencing quality. *De novo* assembly of the clean reads generated 41.56–49.30 million unigenes, among which 27,942 were successfully annotated. The overall GC content averaged 43.53% (Supplementary Table S1). Length distribution analysis revealed that unigenes shorter than 600 bp comprised approximately 88% of the total mapped reads (Supplementary Table S2). These results confirm that the sequencing data are of high quality and suitable for downstream functional analyses. All unigenes were functionally annotated by sequence similarity searches against the Gene Ontology (GO) and Kyoto Encyclopedia of Genes and Genomes (KEGG) databases (Supplementary Table S2).

GO term analysis of the *S. tuberosum* leaf transcriptome revealed that a total of 30 terms were assigned to the biological process category, with “biological process,” “cellular component,” and “molecular function” representing the three primary GO ontologies. Within the cellular component category, 10 terms were significantly represented, among which “membrane,” “lipid droplet,” and “cell part” were the most prominent. Similarly, 10 terms were classified under molecular function, primarily including “oxidoreductase activity” and “catalytic activity” (Supplementary Figure S2A). In addition, 16 KEGG pathways were annotated, with the most enriched being “carbohydrate metabolism,” “signal transduction,” and “biosynthesis of other secondary metabolites” (Supplementary Figure S2B).

A correlation analysis of gene expression levels among samples is shown in Supplementary Figure S3, indicating high intra-group consistency, which supports the reliability of subsequent differential expression analysis. Principal component analysis (PCA) of the six samples further demonstrated a clear separation between virus-infected and control groups, with the first principal component (PC1) explaining 71.44% of the total variance, while the second component (PC2) accounted for 17.83%, primarily reflecting differences within the *S. tuberosum* leaf samples (Figure 2B). These supported the biological relevance of the experimental treatments.

### 3.3 Identification of DEGs in Co-88Responsive to PVS infection

Differentially expressed genes (DEGs) across experimental comparisons were identified using DESeq2 based on fragments per kilobase of FPKM values. For PVS-infected (designated as “V”) and mock-inoculated (designated as “CK”) comparison, 588 DEGs were detected: 161 upregulated and 427 downregulated (Supplementary Figure S1). These results indicate significant alterations in gene expression profiles in potato leaves following PVS infection. The large number of DEGs identified may reflect the complex genetic background of *S. tuberosum*.

Notably, 161 upregulated DEGs were uniquely identified in this comparison, suggesting their potential involvement in antiviral defense mechanisms. Based on leaf-specific DEG expression patterns, all DEGs were hierarchically clustered into two distinct groups, visualized via heatmaps (Supplementary Figure S4). However, the identification of numerous PVS-responsive DEGs—coupled with the lack of a complete genome sequence for this complex polyploid species—complicates direct pinpointing of resistance genes. To address this, we employed bioinformatics resources including Gene Ontology (GO) and Kyoto Encyclopedia of Genes and Genomes (KEGG) analyses, which facilitate the prioritization of biologically relevant pathways and candidate DEGs.

KEGG functional annotation revealed that PVS resistance-related DEGs were primarily enriched in three major categories: “molecular function,” “cellular component,” and “biological process” (Supplementary Figure S5). Further KEGG pathway enrichment analysis identified 16 commonly enriched pathways across comparisons, with “plant hormone signal transduction” and the “MAPK signaling pathway-plant” emerging as the most significantly overrepresented (Figure 2D). The high enrichment of these two pathways in the *Solanum tuberosum* cv. Cooperation 88 comparison suggests they are likely central to potato resistance against PVS.

Compared to the V vs. CK dataset (Figure 2D), five additional pathways were uniquely enriched: “sulfur metabolism,” “flavonoid biosynthesis,” “amino sugar and nucleotide sugar metabolism,” “phenylpropanoid biosynthesis,” and “ascorbate and aldarate metabolism” (Figure 2D). These five pathways are all potentially associated with PVS resistance. Collectively, these findings suggest that DEGs within the “flavonoid biosynthesis” and “plant hormone signal transduction” pathways may play particularly critical roles in mediating *S. tuberosum*’s resistance to PVS.

### 3.4 Candidate flavonoid DAMs positively dominate the resistance of Co-88 against PVS

**Figure 3:**
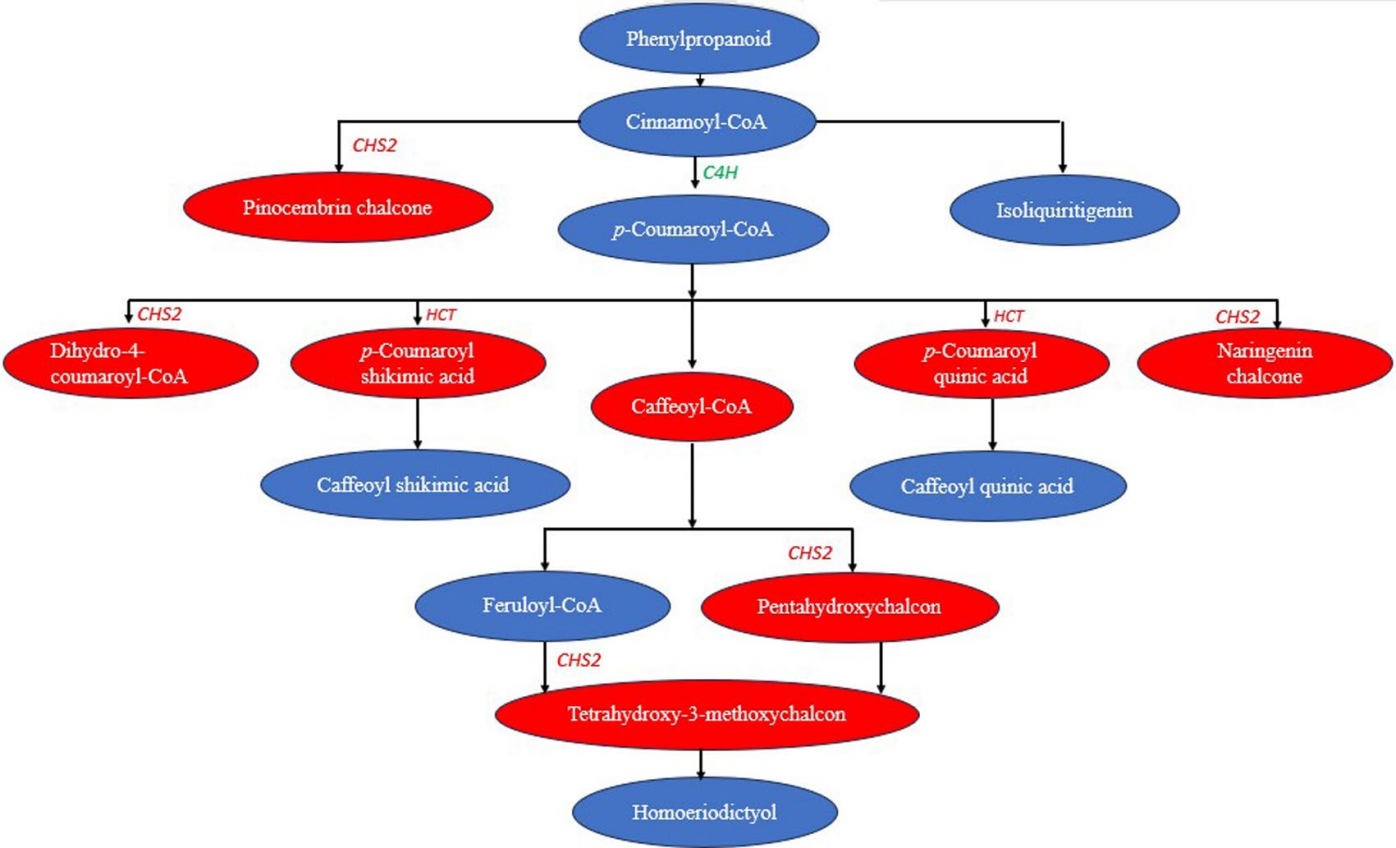
Flavonoid metabolic cycle diagram. Red: up transcription factor; Green: down transcription factor; Blue: not different transcription factors.

Differential accumulated metabolites (DAMs) in Co-88were predominantly enriched in the biosynthesis of plant secondary metabolites, particularly phenylpropanoids and flavonoids. The phenylpropanoid pathway mediates the biogenesis of diverse phenolic polymers, including flavonoids and phenolic acids—metabolites well-documented as critical structural and signaling molecules in plant development and defense (Yadav V. et al., 2020). In this study, flavonoid and phenolic acid DAMs represented the most highly enriched metabolite classes. Among the upregulated DAMs, four were associated with the biosynthesis of other secondary metabolites, and four with amino acid metabolism (Figure 2G). These results indicate that PVS infection induces elevated accumulation of flavonoids and phenolic acids in potato (Figure 3), aligning with prior reports demonstrating that dramatic increases in flavonoids and phenolic compounds are integral to disease resistance in plant-virus interactions. Highlight 10 defense related DEGs and clarify their expression patterns and antiviral roles (Supplementary Table S6).

Flavonoid biosynthesis constitutes a key downstream branch of phenylpropanoid metabolism and has been extensively studied for its protective roles against environmental stresses (Yadav S. et al., 2020). Here, flavonoid DAMs were the most abundant, comprising 4 upregulated and 12 downregulated species (Figure 2G). KEGG pathway analysis further revealed significant enrichment of flavonoid metabolism in PVS-infected samples compared to control (CK) plants (Figure 2D). Collectively, these findings suggest that flavonoid metabolism may play a dominant positive role in mediating *S. tuberosum* resistance to PVS.

### 3.5 Metabolome analysis of Co-88 leaves in response to PVS infection

**Figure 4:**
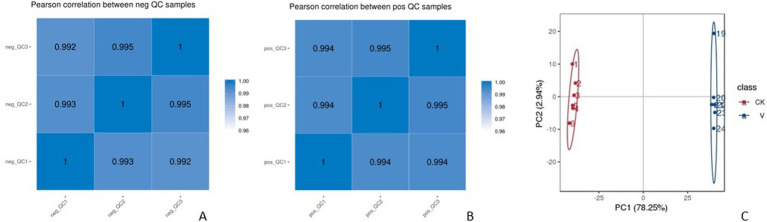
**(A)** Positive ionization QC quality; **(B)** Negative ionization QC quality; **(C)** PCA showed clustering of quality control (QC) samples (the number is the samples number).

A widely untargeted metabolome analysis was performed on *S. tuberosum* leaf samples collected at 28 days post-inoculation (dpi) with PVS (designated as “V”) or mock-inoculated (designated as “CK”). A total of 27,942 metabolites were detected across all samples, with a heatmap visualization revealing distinct hierarchical clustering of samples by treatment (Figure 2B). Principal component analysis (PCA) showed tight clustering of quality control (QC) samples, indicating high reproducibility and stability of the metabolomic profiling (Figures 4A–C).

Differential metabolites were identified using thresholds of variable importance in projection (VIP) > 1.0, fold change (FC) > 1.5 or FC < 0.667, and *p*-value < 0.05. In the PVS vs. CK comparison, 1,313 DAMs were detected in positive ion mode (613 upregulated, 700 downregulated), and 880 DAMs in negative ion mode (704 upregulated, 176 downregulated) (Supplementary Table S5). A heatmap of DAMs confirmed substantial metabolomic alterations in *S. tuberosum* leaves following PVS infection (Figure 2C).

DAMs were categorized into 15 biochemical classes, with KEGG pathway analysis highlighting enrichment in “biosynthesis of secondary metabolites,” “global and overview maps,” “metabolism of terpenoids and polyketides,” “metabolism of other amino acids,” “nucleotide metabolism,” “metabolism of cofactors and vitamins,” and “amino acid metabolism” (Figure 2E, 2F). Notably, metabolites involved in “phenylpropanoid biosynthesis” and “flavonoid biosynthesis” exhibited marked accumulation, consistent with transcriptomic data on gene expression levels.

### 3.6 Integrated transcriptome-metabolome analysis of Co-88 leaves in response to PVS infection

To dissect the relationship between differentially expressed genes (DEGs) and differential accumulated metabolites (DAMs) in PVS-infected Co-88 leaves, a coexpression network analysis was conducted using transcriptomic data. Combined KEGG pathway analysis of DEGs and DAMs revealed consistent expression patterns in several pathways, including “flavonoid biosynthesis,” “glycerophospholipid metabolism,” “phenylpropanoid biosynthesis,” and “tryptophan metabolism” (all with *p* < 0.05) (Figure 3). Among these, only the DEGs and DAMs associated with “flavonoid biosynthesis” showed extremely significant concordance (*p* < 0.01), further validating the conclusion that flavonoid biosynthesis is a key positive regulator of resistance to PVS infection.

### 3.7 Profiles of genes associated with flavonoid biosynthesis of *Solanum tuberosum* leaves in response to PVS infection

Transcriptome analysis revealed that flavonoid metabolism—particularly the pathways leading to flavones and flavonols—plays a significant role in the resistance of Co-88 leaves following PVS infection. To further elucidate this, the biosynthetic routes of flavones and flavonols in Co-88 leaves in response to PVS infection were mapped in detail using the KEGG database (Figure 3). Flavonoid biosynthesis originates from the phenylpropanoid pathway, in which three key enzymes—phenylalanine ammonia-lyase (PAL), trans-cinnamate-4-monooxygenase (C4H), and 4-coumarate-CoA ligase (4CL)—catalyze the conversion of phenylalanine into cinnamic acid and *p*-coumaroyl-CoA. This step marks a critical branch point, after which the pathway diverges into the phenylalanine metabolic route and the flavonoid metabolic route (Dixon et al., 2002). The activity levels of these enzymes closely matched the expression patterns of their corresponding genes, suggesting coordinated regulation that may lead to synchronized accumulation of their catalytic products. The biosynthesis of each flavonoid metabolite involves a complex network; therefore, detailed investigation of this network is essential when aiming to enhance or utilize specific target metabolites.

Flavonoid biosynthesis is a highly intricate metabolic process regulated by multiple enzymes, whose composition and activity may differ depending on plant species and tissue types. Previous studies have documented flavonoid biosynthesis in *S. tuberosum* leaves, annotating several relevant genes such as 4CL, CHS, CHI, F3H, DFR, ANS, ANR, and FLS (Bao et al., 2022; Li et al., 2025). However, the biosynthetic pathway characterized in the present study provides new insights that could facilitate targeted manipulation of flavonoid biosynthesis to enhance resistance against PVS in *S. tuberosum*. Further investigation into this area is warranted.

Given the extensive knowledge accumulated regarding the genetics, biochemistry, and molecular biology of flavonoid metabolism in various plant species, it is both practical and promising to harness this pathway for improving disease resistance. The primary focus of our current research was to identify key genes associated with flavonoid biosynthesis that contribute to PVS resistance in *S. tuberosum*. These findings offer valuable guidance for future efforts to engineer enhanced resistance in this crop through genetic modification strategies.

### 3.8 RT-qPCR validation of DEGs associated with flavonoid biosynthesis in Co-88

**Figure 5:**
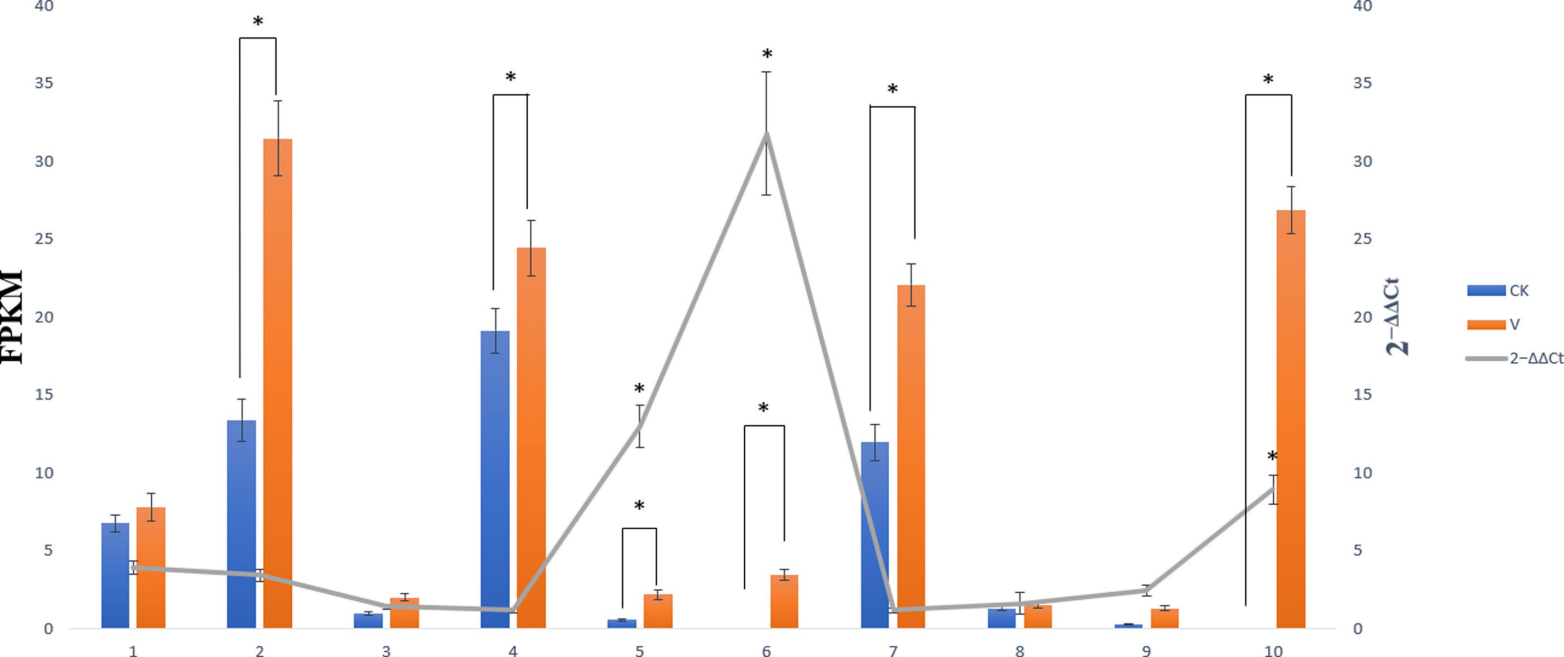
Metabolome result of *S. tuberosum* leaves responsive to PVS infection. 1–10: (1) 3-ketoacyl-CoA synthase 6-like (GeneID: 102579394); (2) Chalcone-flavanone isomerase family protein (GeneID: 102583606); (3) CAP protein (GeneID: 102590095); (4) hydroxycinnamoyl-CoA shikimate/quinate hydroxycinnamoyl transferase (GeneID: 102577662) (5) Chalcone and stilbene synthase family protein (GeneID: 102590425); (6) Chalcone and stilbene synthase family protein (GeneID: 102595537) (7) RPM1 interacting protein (GeneID: 102596147); (8) Enoyl-CoA hydratase/isomerase family (GeneID: 102587367); (9) Peroxidase superfamily protein (GeneID: 102583777); (10) phytochrome interacting factor (GeneID: 102588413).

To assess the reliability and reproducibility of the transcriptome analysis, we conducted Reverse Transcription-quantitative PCR (RT-qPCR) on a panel of differentially expressed genes (DEGs) associated with flavonoid biosynthesis. Based on their involvement in the flavonoid metabolic pathway, ten candidate DEGs were selected for RT-qPCR validation (Figure 5). The results revealed that the expression patterns of these ten genes were generally consistent with the FPKM values derived from RNA-Seq analysis, and also correlated well with the trends observed in the metabolomic data. Following PVS infection, the expression levels of key flavonoid biosynthetic genes were significantly upregulated in resistant leaves compared to the control group. Moreover, the expression profiles obtained from both RNA-Seq and RT-qPCR were largely congruent, supporting the high reliability of the transcriptomic and metabolomic datasets generated in this study.

## 4 Discussion

In plant-pathogen interactions, secondary metabolites act as key signaling molecules mediating plant defense responses (Li et al., 2020; Jiang et al., 2025). The signal transduction of secondary metabolites can alter ion flow across cell membranes and induce calcium ion release, thereby activating defense gene expression and regulating secondary metabolic pathways to synthesize phytoalexins, which combat pathogen invasion and colonization (Boller et al., 2009). Most phytoalexins are synthesized via the phenylpropanoid pathway, including flavonoids, terpenoids, alkaloids, and phenolic acids (Ahuja et al., 2012).

In this study, transcriptome analysis revealed that differentially expressed genes (DEGs) were significantly enriched in the “plant hormone signal transduction” and “flavonoid biosynthesis” pathways, suggesting that potato resistance to Potato virus S (PVS) may be regulated by plant hormones through flavonoid metabolism. Metabolomic data further indicated that PVS infection induced significant accumulation of flavonoids and phenolic acids in potato leaves, consistent with previous reports that flavonoids enhance disease resistance in plant-virus interactions. For example, phenolic acid accumulation improves citrus resistance to Citrus Tristeza Virus (Munir et al., 2019); the phenylpropanoid pathway has also been shown to enhance tobacco resistance to Tobacco Mosaic Virus (Shadle et al., 2003), tomato resistance to Tomato Yellow Leaf Curl Virus (TYLCV) (Yao et al., 2019), and watermelon resistance to Cucumber Green Mottle Mosaic Virus (CGMMV) (Liu et al., 2023).

Flavonoids are among the most extensively studied secondary metabolites in plant defense systems. When subjected to biotic or abiotic stress, plants activate all or part of their defense networks, exhibiting specific metabolic response patterns (Górniak et al., 2018). This study hypothesizes that phenolic acids, flavones, and flavonols may play important roles in potato resistance to PVS. Additionally, viruses not only interfere with primary metabolism but also regulate host secondary metabolism to alter defense mechanisms. For instance, Grapevine Red Blotch Virus (GRBV) suppresses the phenylpropanoid pathway and its derivatives, weakening the host’s ability to produce antiviral compounds (Rumbaugh et al., 2022); other studies, however, indicate that plant viruses can induce phenolic compound synthesis in response to infection (Rabie et al., 2024; Rashad et al., 2020; Abdelkhalek et al., 2022). These findings reveal the dynamic and complex interactions between viruses and host plants at the level of flavonoid metabolism.

Flavonoids are a class of compounds formed by two benzene rings connected by a three-carbon chain, primarily including flavones, flavonols, flavanones, dihydroflavonols, flavanols, chalcones, and dihydrochalcones (Liu et al., 2021). This study identified ten differentially accumulated metabolites (DAMs) responsive to PVS infection, belonging to ten subclasses. KEGG enrichment analysis showed that changes in the “flavonoid biosynthesis” and “flavone and flavonol biosynthesis” pathways were more pronounced than those in the “isoflavonoid biosynthesis” pathway. Flavonoid biosynthesis is the upstream initial pathway for flavonoid synthesis, with downstream branches including “flavone and flavonol biosynthesis,” “isoflavonoid biosynthesis,” and “anthocyanin biosynthesis” (Górniak et al., 2018; Bao et al., 2022). Therefore, we speculate that flavonoid metabolism—particularly the synthesis of flavones and flavonols—plays a dominant role in potato resistance to PVS.

Transcriptome analysis revealed significant changes in DEGs within the “plant hormone signal transduction” pathway, but metabolomics did not detect significant DAMs in this pathway. This pathway primarily involves eight classical plant hormones: auxin, cytokinin, gibberellin, abscisic acid, ethylene, brassinosteroid, jasmonic acid, and salicylic acid. Notably, we detected one downregulated metabolite—serotonin (which shares partial functional similarity with auxin). Multiple studies have shown that plant flavonoid biosynthesis is regulated by plant hormones (Maridueña-Zavala et al., 2025a,b; Sun et al., 2019; Wang et al., 2022). For example, after papaya infection with Babaco Mosaic Virus (BabMV), phenylalanine levels and phenylalanine ammonia-lyase (PAL) activity increased, explaining the rise in phenolic and flavonoid levels; Yang et al. (2019) found that exogenous abscisic acid and melatonin significantly increased flavonol accumulation in grape berries and upregulated flavonoid biosynthesis-related gene expression. This suggests that the regulatory effects of plant hormones on flavonoid synthesis vary depending on plant species and virus type. Although this study did not directly obtain evidence of plant hormone regulation of flavonoid synthesis, the potential regulatory roles of 3-indolepropionic acid and serotonin in potato flavonoid metabolism warrant further investigation, which could help clarify the role of flavonoid metabolic regulation in potato resistance to PVS.

Since flavonoid biosynthesis originates from the phenylpropanoid pathway, we conducted co-expression network analysis on DEGs and DAMs annotated to these two pathways. The results showed that metabolites in the phenylpropanoid biosynthesis pathway exhibited both positive and negative regulatory relationships with related genes, while some metabolites in flavonoid biosynthesis were also regulated by key genes. Additionally, shared DEGs and DAMs participating in both phenylpropanoid and flavonoid biosynthesis were observed in the network, which may be related to the phenylpropanoid pathway serving as the upstream route for flavonoid synthesis (Wang et al., 2017).

In summary, potato resistance to PVS may be closely associated with the coordinated expression and regulation of DEGs and DAMs related to flavonoid biosynthesis.

## 5 Conclusion

Integration of transcriptomic and metabolomic analyses revealed that, compared with healthy leaves, genes and metabolites associated with phenylpropanoid metabolism (especially the flavonoid biosynthesis pathway) were significantly enriched in potato leaves infected with Potato virus S (PVS). This suggests that flavonoid metabolism may play a crucial regulatory role in the interaction between potato and PVS. Reverse Transcription-quantitative PCR (RT-qPCR) results further validated this finding. The above results indicate that the flavonoid biosynthesis pathway in potato plays a key role in responding to PVS infection. This study not only helps to deepen the understanding of the interaction mechanism between PVS and potato but also provides a theoretical basis for the breeding and utilization of disease-resistant potato varieties.

## Data Availability

The original contributions presented in the study are included in the article/Supplementary material, further inquiries can be directed to the corresponding authors.
