# Supplementary figures and images for: Integrated analysis of transcriptome sequencing and metabolomics provides insights into the molecular response of Solanum tuberosum cv. Cooperation 88 to Potato virus S
Source: Front Microbiol. 2026 Jul 15;17:1828392. doi: 10.3389/fmicb.2026.1828392 (PMC13416330; doi:10.3389/fmicb.2026.1828392)

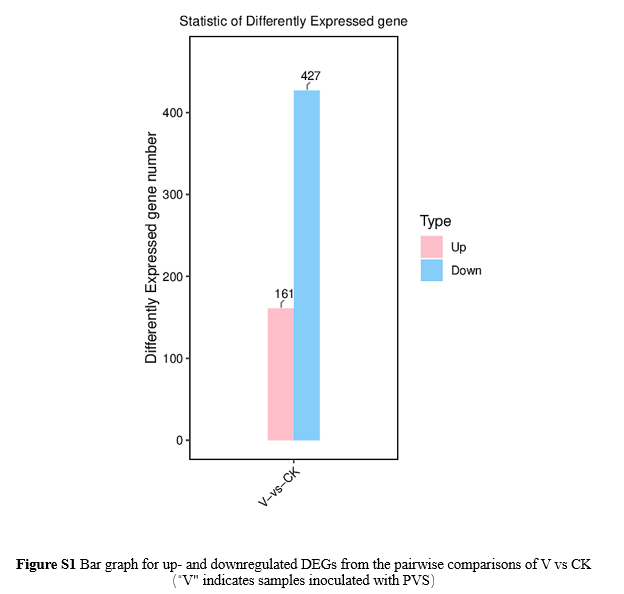

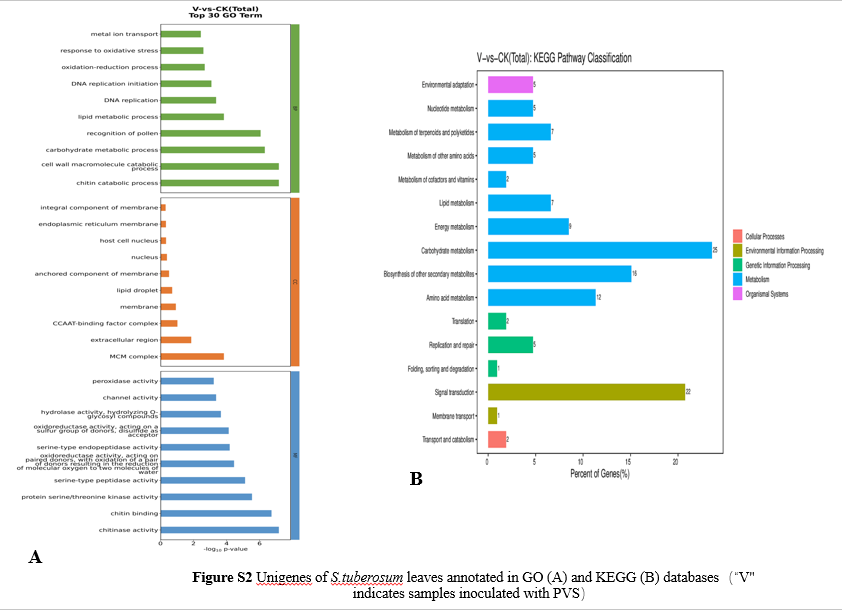

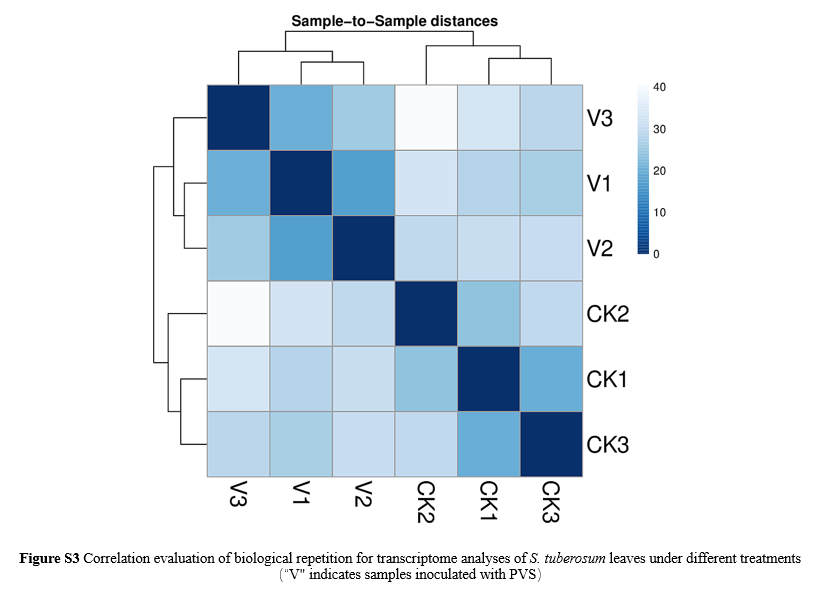

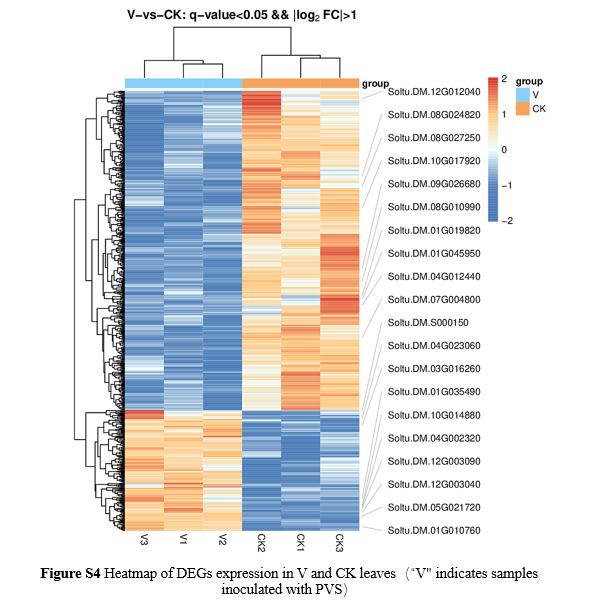

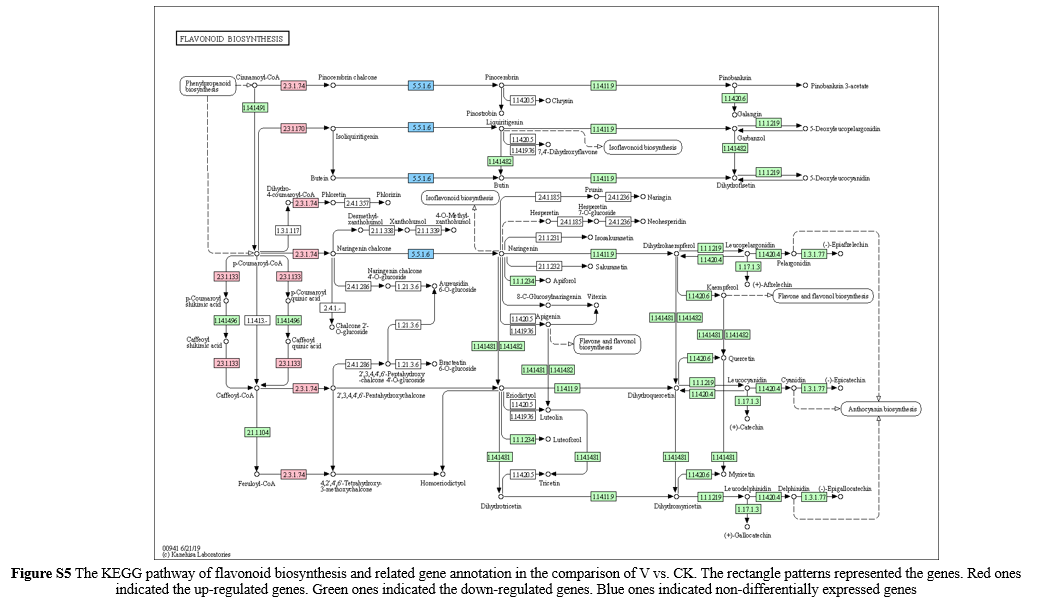

Supplement: Supplementary file 2 [file Table_2.DOCX]
